# Prevalence of antibiotic-resistant Gram-negative bacteria having extended-spectrum β-lactamase phenotypes in polluted irrigation-purpose wastewaters from Indian agro-ecosystems

**DOI:** 10.3389/fmicb.2023.1227132

**Published:** 2023-08-07

**Authors:** Achhada Ujalkaur Avatsingh, Shilpa Sharma, Shilippreet Kour, Yukta Arora, Sheetal Sharma, Divya Joshi, Prem Prashant Chaudhary, Kahkashan Perveen, Mohab Amin Kamal, Nasib Singh

**Affiliations:** ^1^Department of Microbiology, Akal College of Basic Sciences, Eternal University, Baru Sahib, Sirmaur, Himachal Pradesh, India; ^2^Department of Microbiology, College of Basic Sciences and Humanities, GBPUA&T, Pantnagar, Uttarakhand, India; ^3^Epithelial Therapeutics Unit, National Institute of Allergy and Infectious Diseases, National Institutes of Health, Bethesda, MD, United States; ^4^Department of Botany & Microbiology, College of Science, King Saud University, Riyadh, Saudi Arabia; ^5^Environmental Engineering, Civil Engineering Department, College of Engineering, King Saud University, Riyadh, Saudi Arabia

**Keywords:** wastewater, antibiotics, pollution, antibiotic-resistant bacteria, *Enterobacterales*, extended-spectrum beta-lactamases, agro-ecosystems

## Abstract

Antibiotic resistance in bacteria has emerged as a serious public health threat worldwide. Aquatic environments including irrigation-purpose wastewaters facilitate the emergence and transmission of antibiotic-resistant bacteria and antibiotic resistance genes leading to detrimental effects on human health and environment sustainability. Considering the paramount threat of ever-increasing antibiotic resistance to human health, there is an urgent need for continuous environmental monitoring of antibiotic-resistant bacteria and antibiotic resistance genes in wastewater being used for irrigation in Indian agro-ecosystems. In this study, the prevalence of antibiotic resistance in Gram-negative bacteria isolated from irrigation-purpose wastewater samples from Sirmaur and Solan districts of Himachal Pradesh was determined. Bacterial isolates of genera *Escherichia, Enterobacter, Hafnia, Shigella, Citrobacter*, and *Klebsiella* obtained from 11 different geographical locations were found to exhibit resistance against ampicillin, amoxyclav, cefotaxime, co-trimoxazole, tobramycin, cefpodoxime and ceftazidime. However, all the isolates were sensitive to aminoglycoside antibiotic gentamicin. *Enterobacter* spp. and *Escherichia coli* showed predominance among all the isolates. Multidrug-resistance phenotype was observed with isolate AUK-06 (*Enterobacter* sp.) which exhibited resistant to five antibiotics. Isolate AUK-02 and AUK-09, both *E. coli* strains showed resistant phenotypes to four antibiotics each. Phenotypic detection revealed that six isolates were positive for extended-spectrum β-lactamases which includes two isolates from *Enterobacter* spp. and *E. coli* each and one each from *Shigella* sp. and *Citrobacter* sp. Overall, the findings revealed the occurrence of antibiotic resistant and ESBL-positive bacterial isolates in wastewaters utilized for irrigation purpose in the study area and necessitate continuous monitoring and precautionary interventions. The outcomes of the study would be of significant clinical, epidemiological, and agro-environmental importance in designing effective wastewater management and environmental pollution control strategies.

## 1. Introduction

Antibiotics are important anti-infective agents which have been used since the 20th century for the treatment of human infections (Hutchings et al., 2019; Walesch et al., 2023). The β-lactam antibiotics are clinically important antimicrobial medicines and have remained the first-line chemotherapeutic intervention against Gram-positive and Gram-negative bacteria since the 1950s (Hutchings et al., 2019; Soliman et al., 2023). Bacterial resistance to β-lactams has increased substantially in past few decades (Jani et al., 2021; Zaatout et al., 2021; Mutuku et al., 2022; Tan et al., 2023). However, their irrational, injudicious, and excessive use is on steady rise which not only worsen the issue of antibiotic resistance but also resulted in their accumulation in the environment as micro-pollutant (Du and Liu, 2012; Fazaludeen Koya et al., 2022; Gitter et al., 2023). The emergence of antibiotic resistance has threatened the effective treatment of microbial infections (Sanz-García et al., 2023). According to World Health Organization, the majority of pathogenic Gram-negative bacteria, especially those of the family *Enterobacteriaceae,* are included in the critical-priority group and are represented by multidrug resistant (MDR) bacteria commonly encountered in healthcare settings (Janda and Abbott, 2021; Dantas Palmeira et al., 2022). Further, antibiotic resistance is recognized as one of the top ten threats to public health globally (World Health Organization, 2023). In 2019, an estimated 4·95 million deaths were attributed to antibiotic resistance in bacterial pathogens such as *E. coli*, *Klebsiella pneumoniae*, *Pseudomonas aeruginosa, Acinetobacter baumannii,* and *Streptococcus pneumoniae* (Antimicrobial Resistance Collaborators, 2022).

Among various mechanisms by which bacteria acquired antibiotic resistance, the production of β-lactamases (EC 3.5.2.6) which cleaves β-lactam antibiotics, is considered the most significant from clinical perspective (Sawa et al., 2020; Castanheira et al., 2021; Noster et al., 2021; Zaatout et al., 2021). These enzymes are categorized into four classes according to their amino acid sequence and catalytic mechanisms (Bush, 2023). Class A β-lactamases are represented by narrow-spectrum β-lactamases (TEM-1, TEM-2, and SHV-1), extended-spectrum β-lactamases also called ESBLs (CTX-M, SHV-2, VEB-1) and serine carbapenemases which includes KPC-1 and SME-1 (Bush and Bradford, 2020; Castanheira et al., 2021; Bush, 2023; Cho et al., 2023). ESBLs are highly diverse, clinically important, and constitutively expressed antibiotic-degradative enzymes capable of hydrolyzing penicillins, cephalosporins (first-, second-, and third-generations), and monobactams (Livermore, 2008; Castanheira et al., 2021; Cho et al., 2023). ESBLs have been reported from several members of *Enterobacterales*, predominantly from genera *Escherichia, Enterobacter, Citrobacter,* and *Klebsiella* (Janda and Abbott, 2021; Cho et al., 2023). Worldwide, the most prevalent ESBL groups are CTX-M-1, CTXM-2, CTX-M-8, CTX-M-9, and CTX-M-25 with *bla_CTX-M-15_* genotype being the predominant (Bush and Bradford, 2020; Castanheira et al., 2021; Bush, 2023; Cho et al., 2023). The ESBL-encoding genes are usually carried by mobile genetic elements *viz.* plasmids, insertion sequences, integrons, integrative conjugative elements, mobile integrative conjugative elements, transposons and prophages, which have facilitated their wide dissemination to other bacterial species through transformation, conjugation, and transduction (Partridge et al., 2018; Castanheira et al., 2021; Zaatout et al., 2021).

Wastewaters from municipal corporations, hospitals, pharmaceutical companies, animal husbandry, and poultry farms contain a diverse array of antibiotic residues, antibiotic-resistant bacteria (ARB) and antibiotic-resistant genes (ARGs) (Leiva et al., 2021; Uluseker et al., 2021; Larsson and Flach, 2022; Gitter et al., 2023). The combination of all these factors in aquatic environments allows rapid genetic exchange of ARGs from pathogenic to non-pathogenic bacterial strains leading to their genetic evolution and subsequent dissemination to human communities and animals (Liguori et al., 2022; Matthiessen et al., 2022; Muntean et al., 2022; Mutuku et al., 2022). Overuse and misuse of antibiotics have resulted in the widespread occurrence of ARB in surface water of rivers, irrigation-wastewater, soil, meat products, vegetables, etc., which possess the risk of dissemination in vulnerable human populations such as children, elderly, and immunocompromised persons (Uluseker et al., 2021; Zaatout et al., 2021; Gitter et al., 2023). The infections caused by multidrug-resistant bacteria can cause high morbidity and mortality, higher treatment costs and longer hospitalization duration. Over the years, the use of treated wastewater for agricultural purposes have gained widespread acceptance as an alternative irrigation method to relieve pressure on freshwater resources, to increase agricultural production, and to reduce the need for chemical fertilizers (Leiva et al., 2021; Minhas et al., 2022). However, this practice has led to continuous buildup of antibiotic residues in agro-ecosystems. ARB enter the aquatic environments from human and animal waste, fecal matter and hospital discharge where these are able to proliferate due to the availability of the nutrients, antibiotic residues, and inappropriate treatment and disinfection practices. Transmission of ARB from wastewaters into agro-ecosystems represents a serious ecological and public health concern and requires immediate interventions (Bhagat et al., 2020; Helmecke et al., 2020; Tucker et al., 2022). The occurrence of ARGs *viz. bla* (*bla*_CTX-M_, *bla*_TEM_), *tet* (*tet*O, *tet*Q, *tet*W), *sul* (*sul*1, *sul*2), and *ermB* has been reported from ARB present in the riverine systems, municipal wastewater treatment plants, pharmaceutical industries effluents, and irrigated soils (Karkman et al., 2018; Grenni, 2022; Cho et al., 2023; Shin et al., 2023). Therefore, there is an urgent need for environmental monitoring of ARB and ARGs in aquatic environments for limiting the transmission of antibiotic resistance.

The prevalence and dissemination of ARB carrying ESBLs genes has been reported from diverse agro-ecosystems worldwide. ESBLs genotypes *bla*_CTX-M-1_, *bla*_CTX-M-15_ and *bla*_CTX-M-14_ were found in *E. coli*, *K. pneumoniae*, *E. hormaechei*, and *C. freundii* from Tunisian farm irrigation water samples (Ben Said et al., 2015). Similarly, agricultural-purpose irrigation water also found to harbor ESBL-positive *E. coli* strain exhibiting *bla*_CTX-M-55_, *bla*_CTX-M-65_ and *bla*_CTX-M-15_ genotypes (Montero et al., 2021). The predominant prevalence of *bla*_CTX-M,_ *bla*_CMY_ and *bla*_SHV_ in water samples was also reported from Nepal and Canada (Subramanya et al., 2021; Anderson et al., 2023). India being the largest consumer of antibiotics and other antimicrobials, witnessed a significant increase in the prevalence of ARB and ESBL-positive *Enterobacterales* in recent years (Farooqui et al., 2018; Jani et al., 2021; Fazaludeen Koya et al., 2022). *Escherichia coli* and other Gram-negative bacteria resistant to β-lactams, flouroquinolones, tetracyclines and other classes of antibiotics have been reported from wastewaters, river waters and wastewater treatment plants from different states of India along with co-prevalence of ESBL genotypes and other Class A β-lactamases including *bla*_CTX-M-15_, *bla*_CTX-M-152_, *bla*_CTX-M-205,_ *bla*_SHV_ and *bla*_TEM_ (Bajaj et al., 2015; Hanna et al., 2020). Environmental monitoring revealed the presence of bacteria resistant to multiple antibiotics as well as *bla*_CTX-M,_ *bla*_TEM_, *bla*_SHV_ and other β-lactamase genotypes in water of river Ganga, Gomti, Yamuna and Hindon (Chaturvedi et al., 2020, 2021). The scientific data on the prevalence of antibiotic-resistant Gram-negative bacteria from irrigation-purpose wastewaters are either limited or lacking from Himachal Pradesh. Therefore, this study was aimed to determine the antibacterial susceptibility profiles of Gram-negative bacteria against β-lactams and other classes of antibiotics and the occurrence of MDR and ESBL phenotypes in these isolates from irrigation-purpose wastewaters utilized in different agro-ecosystems of lower Himalayan regions within Himachal Pradesh from Northern India.

## 2. Materials and methods

### 2.1. Study area

**Figure 1:**
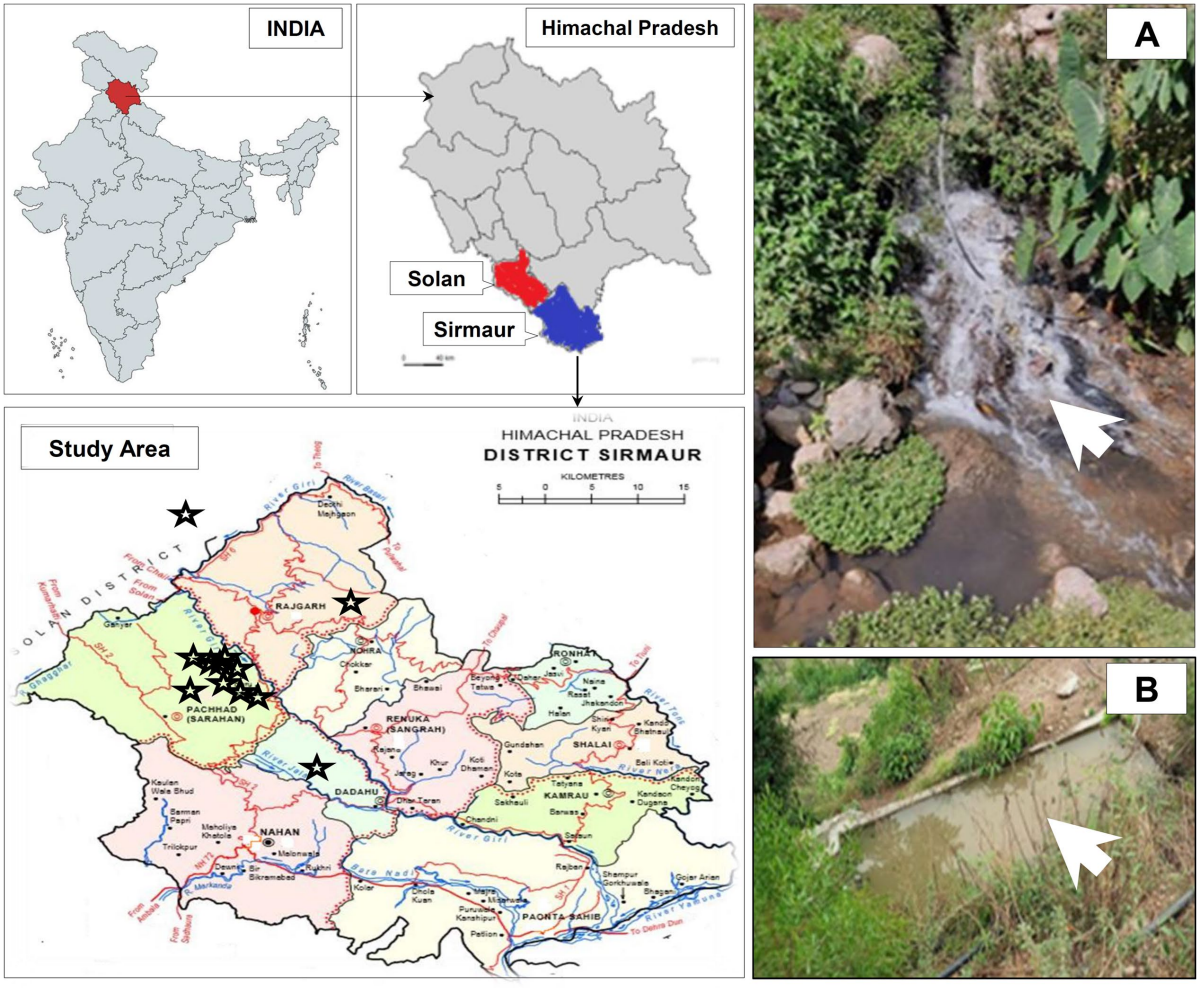
Geographical location of the study area and sampling sites in Sirmaur and Solan districts of Himachal Pradesh, India. The sampling sites are indicated by star symbols. The maps were prepared using https://www.mapchart.net/india.html and https://gadm.org/maps/. Panel **(A**, **B)** represents two of the sampling sites from where the wastewater samples were collected during the study.

The present study was carried out from January, 2022 to July, 2022 in Sirmaur and Solan districts located in the outer Himalayas (Shivalik range) of Himachal Pradesh, India (Figure 1).

### 2.2. Wastewater sample collection

The irrigation-purpose wastewater samples (50 mL) in triplicate were collected (from January, 2022 to July, 2022) in sterile polystyrene screw-capped test tubes from each sampling site as mentioned in Figure 1 and Table 1, kept in ice and immediately transported to the laboratory for bacteriological analysis under aseptic conditions. All necessary safety guidelines and precautions were followed during the sample collection, transportation, analysis and disposal steps.

**Table 1:** List of sampling sites, geographical locations and bacteria isolated from irrigation-purpose wastewaters from lower Himalayan agro-ecosystems located in Himachal Pradesh, India.

S.No.	Sampling site	Tehsil and District	Sample Source	Gram’s reaction	Isolate’s Code
1.	Baru Sahib	Pachhad, Sirmaur	Irrigation-wastewater	Gram-negative	AUK-01 AUK-02
2.	Kakli	Pachhad, Sirmaur	Irrigation-wastewater	Gram-negative	AUK-03
3.	Lana Machher	Pachhad, Sirmaur	Irrigation-wastewater	Gram-negative	AUK-04
4.	Kheri	Pachhad, Sirmaur	Irrigation-wastewater	Gram-negative	AUK-05
5.	Solan City	Solan, Solan	Irrigation-wastewater	Gram-negative	AUK-06
6.	Dadahu	Dadahu, Sirmaur	Irrigation-wastewater	Gram-negative	AUK-07
7.	Rajgarh Town	Rajgarh, Sirmaur	Irrigation-wastewater	Gram-negative	AUK-08
8.	Soda Dhayari	Pachhad, Sirmaur	Irrigation-wastewater	Gram-negative	AUK-09
9.	Bagroti	Pachhad, Sirmaur	Irrigation-wastewater	Gram-negative	AUK-10
10.	Lana Bhalta	Pachhad, Sirmaur	Irrigation-wastewater	Gram-negative	AUK-11
11.	Neri Nawan	Pachhad, Sirmaur	Irrigation-wastewater	Gram-negative	AUK-12

### 2.3. Isolation and characterization of gram-negative bacteria

Gram-negative bacteria were isolated from wastewater samples on Eosin Methylene Blue agar (HiMedia, India). About 100 μL of wastewater sample was spread on selective agar plates in triplicate and incubated at 35–37°C for 24 h as per the methods described earlier (Chaturvedi et al., 2021; Sivaraman et al., 2021). Morphologically distinct colonies based on appearance, color size, margins, texture, etc. were picked and streaked on selective agar plates for bacterial identification using Gram staining kit (HiMedia, India) and biochemical tests kits (HiMedia, India) according to Bergey’s Manual of Systematic Bacteriology/Determinative Bacteriology, and manufacturer’s instructions.

### 2.4. Antibiotic susceptibility testing

Bacterial isolates were evaluated for antibiotic susceptibility profile by Kirby-Bauer disk diffusion method according to standard published methods and Clinical Laboratory Standards Institute (CLSI) guidelines (Drieux et al. 2008; CLSI 2018, 2020). *Escherichia coli* ATCC 25922 (HiMedia, India) was used as a control. The antibiotics and antibiotics/inhibitor combination were obtained from HiMedia, Mumbai (India). Antimicrobial agents belonging to class penicillins, cephalosporins, aminoglycosides, folate pathway antagonists, and β-lactam/inhibitor combinations were tested. The following antibiotics were evaluated: ampicillin (AMP; 10 μg), amoxyclav (AMC; 30 μg), cefotaxime (CTX; 30 μg), co-trimoxazole (COT; 25 μg), gentamicin (GEN; 10 μg), tobramycin (TOB; 10 μg), cefpodoxime (CPD; 10 μg), ceftazidime (CAZ; 30 μg), cefpodoxime/clavulanic acid (CCL; 10/5 μg), ceftazidime/clavulanic acid (CAC; 30/10 μg) and cefotaxime/clavulanic acid (CEC; 30/10 μg). Bacterial isolates were cultured in Mueller-Hinton broth at 35–37°C and the turbidity was adjusted using 0.5 McFarland standard prior to swabbing on Mueller-Hinton agar plates. The antibiotic hexadiscs were placed on agar surface aseptically, followed by incubation at 35–37°C for 16–18 h. The diameter of the zones of inhibition around the disks was recorded to the nearest mm using Antibiotic Zonescale (HiMedia, India). The zones of inhibition diameters were compared with the Performance Standards for Antimicrobial Testing, 2020, Table 2A of M100 document as published by CLSI in order to classify the bacterial isolates either resistant, intermediate, or susceptible to test antibiotics (Drieux et al., 2008); Tan et al., 2023). Bacterial isolate exhibiting resistance against at least three different antibiotics classes was categorized as multidrug-resistant (Magiorakos et al., 2012). The Multiple Antibiotic Resistance (MAR) index was calculated according the formula (Krumperman, 1983) which is as follows: MAR index = number of antibiotics to which an isolate showed resistance/total number of antibiotics evaluated for susceptibility teesting.

**Table 2:** Antibiotic susceptibility testing and resistance phenotypes of Gram-negative bacteria isolated from irrigation-purpose wastewaters from lower Himalayan agro-ecosystems located in Himachal Pradesh, India.

S. No.	Isolate	Genera/species	Antibiotic resistance phenotypes			ESBL phenotype
			Resistant	Intermediate	Sensitive	
1.	AUK-01	*Shigella* sp.	AMP, AMC	CPD	CTX, CPD, GEN, TOB, CAZ	+
2.	AUK-02	*Escherichia coli*	AMP, AMC, CTX, CAZ	CPD	COT, GEN, TOB	+
3.	AUK-03	*Hafnia* sp.	AMP, CTX, CAZ	AMC	COT, GEN, TOB, CPD	−
4.	AUK-04	*Citrobacter* sp.	AMP, AMC	−	CTX, COT, GEN, TOB, CPD, CAZ	+
5.	AUK-05	*Enterobacter* sp.	AMP, AMC, CTX	−	TOB, COT, GEN, CPD, CAZ,	−
6.	AUK-06	*Enterobacter* sp.	AMP, AMC, CTX, COT, TOB	CAZ	GEN, CPD	+
7.	AUK-07	*Enterobacter* sp.	AMP, AMC	CTX	COT, GEN, TOB, CPD, CAZ	−
8.	AUK-08	*Enterobacter* sp.	AMP, AMC, CTX	−	COT, GEN, TOB, CPD, CAZ	+
9.	AUK-09	*E. coli*	AMP, AMC, CTX, CAZ	−	COT, GEN, TOB, CPD	+
10.	AUK-10	*E. coli*	AMP, AMC	−	CTX, COT, GEN, TOB, CPD, CAZ	−
11.	AUK-11	*Klebsiella* sp.	AMP, AMC	−	CTX, COT, GEN, TOB, CPD, CAZ	−
12.	AUK-12	*Enterobacter* sp.	AMP, AMC	−	CTX, COT, GEN, TOB, CPD, CAZ	−

(+) indicate ESBLs positive result, (−) indicates ESBL negative result.

ESBL, Extended-spectrum β-lactamase; AMP, Ampicillin; AMC, Amoxyclav; CTX, Cefotaxime; COT, Co-trimoxazole; GEN, Gentamicin; TOB, Tobramycin; CPD, Cefpodoxime; CAZ, Ceftazidime.

### 2.5. Phenotypic detection of extended-spectrum β-lactamases

Bacterial isolates were tested for ESBLs production by phenotypic confirmatory disk diffusion test (PCDDT) using ESBLs identification kit (HiMedia, India) as per the manufacturer’s instructions. Bacterial isolates were cultured in Mueller-Hinton broth at 35–37°C and the turbidity was adjusted using 0.5 McFarland standard prior to swabbing on Mueller-Hinton agar plates. The disks containing cefotaxime (30 μg) and cefotaxime/clavulanic acid (30/10 μg) were aseptically placed on agar plate containing test bacterial culture at least 24 mm apart and followed by incubation at 35–37°C for 16–18 h. The diameter of the zones of inhibition around the disks was recorded to the nearest mm using Antibiotic Zonescale (HiMedia, India). An increase in the zone of inhibition by ≥5 mm with cefotaxime/clavulanic acid disk was considered ESBL positive result according to the CLSI criteria.

### 2.6. 16S rRNA gene sequencing

Selected bacterial isolates were subjected to 16S rRNA gene sequencing from Chromus Biotech Pvt. Ltd., India using primer set 27F 5´-AGAGTTTGATCMTGGCTCAG-3′ and 1492R: 5´-GGTTACCTTGTTACGACTT-3′ (Palkova et al., 2021). PCR products were sequenced using BigDye terminator cycle sequencing kit (V3.1) in an ABI Prism 3,730 Genetic Analyzer (Applied Biosystems, United States) The nucleotide sequences were subjected to a similarity search using the BLAST algorithm at National Center for Biotechnology Information (NCBI) GenBank Database^1^ and the sequences showing the highest scores were retrieved for further analysis. The 16S rRNA gene sequences of isolates AUK-01, AUK-02, AUK-03 and AUK-04 were deposited in NCBI GenBank database under accession numbers ON968448, ON968449, ON968450, and ON968451, respectively.

### 2.7. Phylogenetic analysis

The 16S rRNA gene sequences were imported to MEGA X v10.2.5 software and phylogenetic trees were constructed on the aligned datasets using the neighbor-joining method (Saitou and Nei, 1987; Tamura et al., 2007). A multiple sequence alignment was performed using the CLUSTAL W program (Thompson et al., 1994) and the data converted to PHYLIP format. All positions containing gaps and missing data were eliminated from the data set (complete deletion option) and branches containing more than 50% gaps were also removed. One sequence from each group was selected as a representative operational taxonomic unit. The phylogenetic tree was constructed by taking the sequences of bacterial strains along with their ten closest type strain matches available in the NCBI database using neighbor joining method with 1,000 bootstrapped replications to estimate evolutionary distance between all pairs of sequences simultaneously.

### 2.8. Statistical analysis

Experiments were performed in triplicates and data were expressed as mean ± standard deviation. Results were analyzed using Microsoft Excel and Graphpad Prism (Dotmatics).

## 3. Results

Wastewaters used for irrigation purpose were collected from 10 different geographical locations in Sirmaur district and one sampling site from Solan district of Himachal Pradesh, India (Table 1). The bacteria growing on selective medium were characterized on the basis of colony features, Gram reaction and biochemical parameters (Supplementary File 1). A total of 12 Gram-negative bacterial isolates were isolated from Baru Sahib (2), Kakli (1), Lana Machher (1), Kheri (1), Solan city (1), Dadahu (1), Rajgarh town (1), Soda Dhayari (1), Bagroti (1), Lana Bhalta (1) and Neri Nawan (1) as shown in Table 1. These isolates were subjected to antibiotic susceptibility testing and detection of ESBL phenotype. The characterization details of the bacterial isolates are provided in Supplementary File 1.

**Figure 2:**
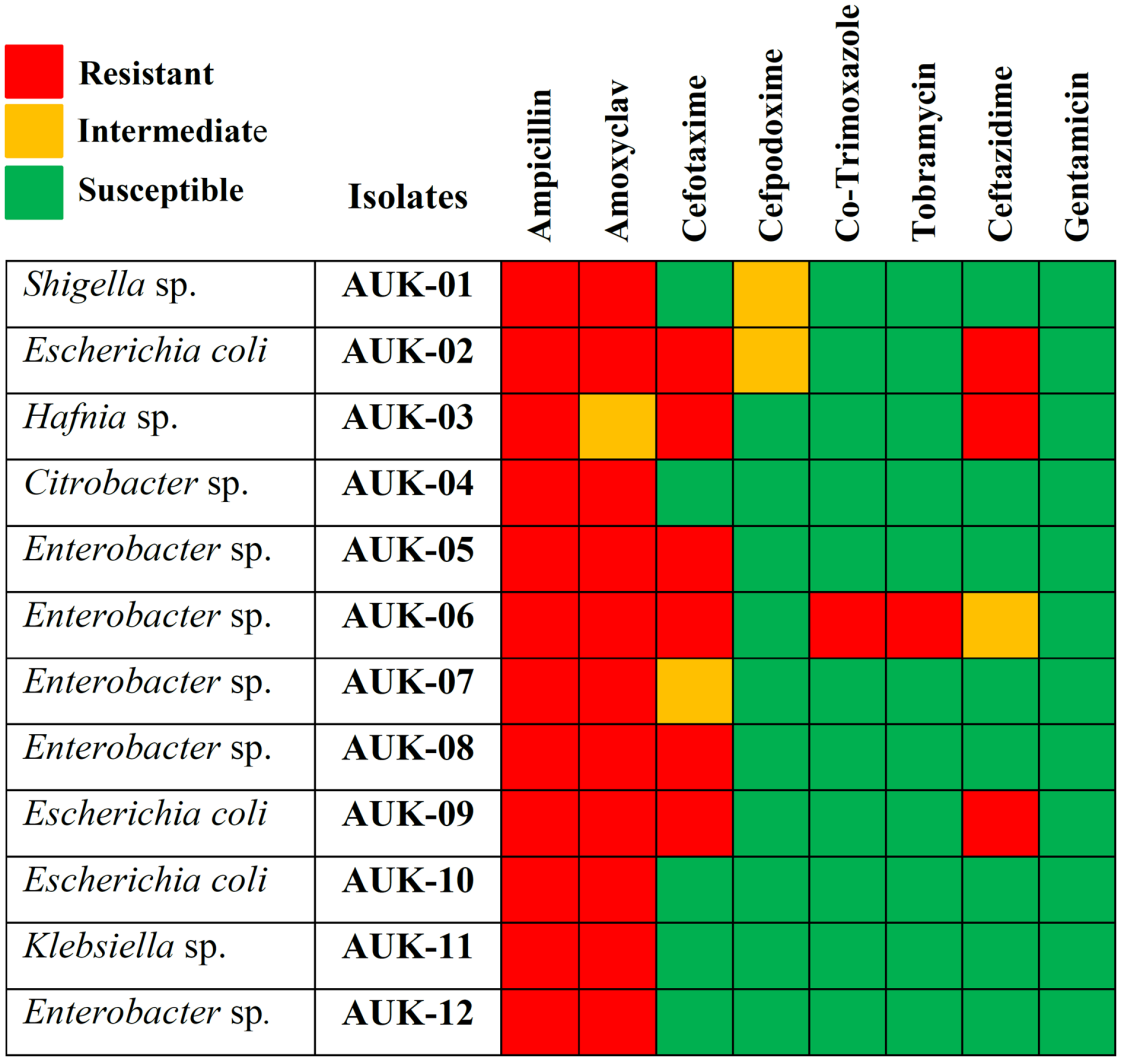
Antibiotic susceptibility heatmap of Gram-negative bacterial isolates as determined by Kirby-Bauer disk diffusion method and interpreted according to CLSI breakpoints. Rows represent bacterial isolates and column represents antimicrobials tested. Red-colored blocks indicate resistance; green blocks indicate susceptible and orange blocks represent intermediate action of the antimicrobial agents.

**Figure 3:**
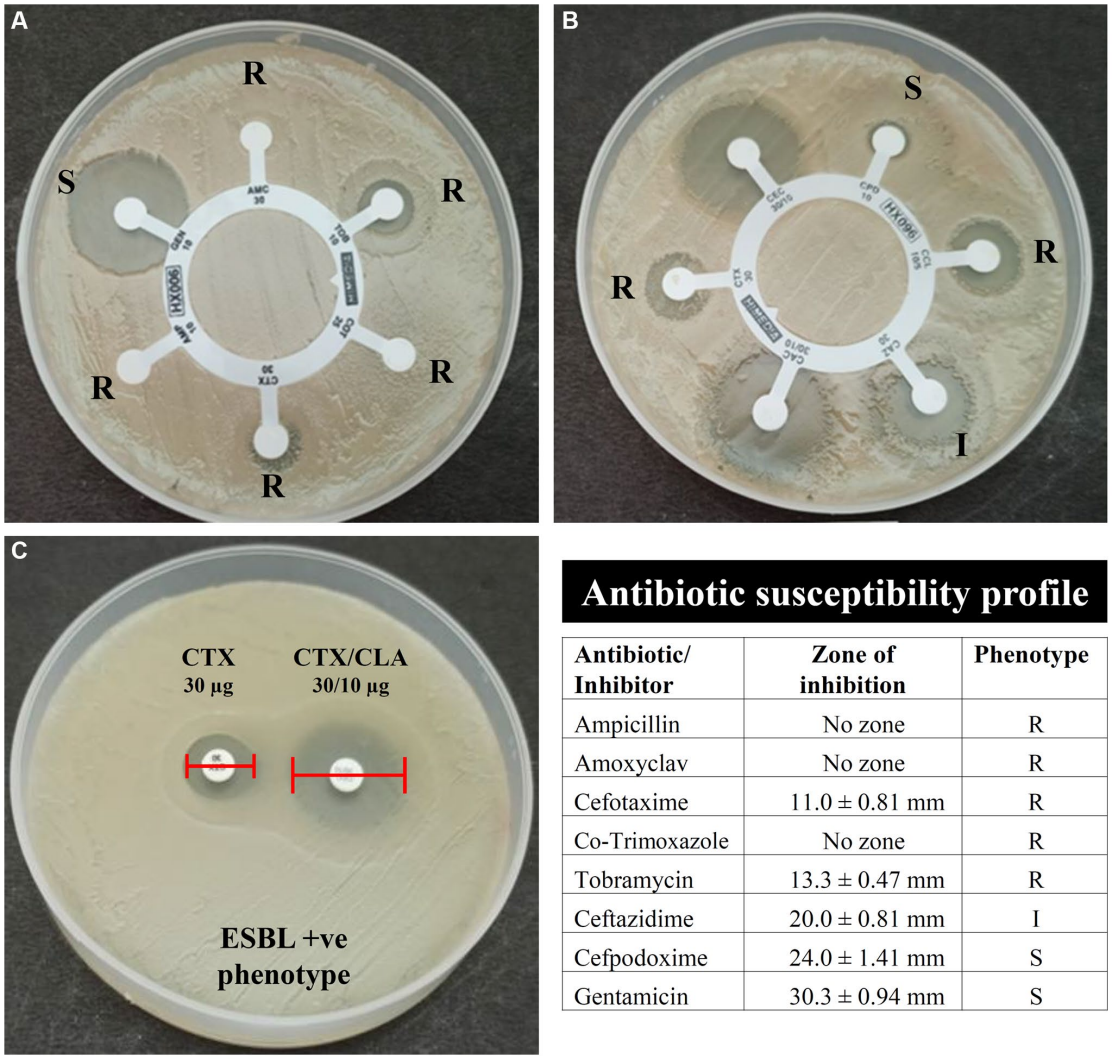
Antibiotic susceptibility and ESBL phenotype of bacterial isolates AUK-06 (*Enterobacter* sp.) as determined by the Kirby-Bauer disk diffusion method. Here, **(A)** indicates susceptibility of isolate AUK-06 toward ampicillin (AMP), amoxyclav (AMC), cefotaxime (CTX), co-trimoxazole (COT), gentamicin (GEN), and tobramycin (TOB); panel **(B)** represent cefpodoxime (CPD), cefpodoxime/CLA (CCL), ceftazidime (CAZ), ceftazidime/CLA (CAC), cefotaxime (CTX), and cefotaxime/CLA (CEC); Phenotypic detection of ESBL production by PCDDT is depicted in **(C)**. The antibiotic susceptibility profile of isolate AUK-06 is shown in tabulated form according to the interpretation criteria mentioned in M100 document, CLSI.

The antibiotic susceptibility testing data are shown in Table 2 and as heatmap in Figure 2. Isolate AUK-06 showed resistant phenotype against five antibiotics *viz.* ampicillin, amoxicillin/clavulanic acid, cefotaxime, co-trimoxozole and tobramycin (Figure 3). It was susceptible to other antibiotics except ceftazidime where it exhibited intermediate phenotype. AUK-02, AUK-03 and AUK-09 were resistant to four antimicrobials (Table 2 and Figure 2). On the other hand, isolates AUK-04, AUK-10, AUK-11 and AUK-12 were susceptible to all the tested antimicrobials except ampicillin and amoxicillin/clavulanic acid combination. AUK-06 was the only isolate that displayed MDR phenotype as it exhibited resistance to at least three classes of antibiotics *viz.*, β-lactams (ampicillin and amoxyclav), aminoglycosides (tobramycin), and cephalosporins (cefotaxime and ceftazidime). As shown in Figure 2, all the bacterial isolates (n = 12) were resistant to ampicillin. In contrast, gentamicin exhibited antimicrobial action against all the isolates, as none were resistant to this antibiotic. In terms of susceptibility toward each of the tested antibiotic, all of the bacterial isolates were resistant to ampicillin whereas 11 were resistant to amoxyclav and six to cefotaxime. Other tested antibiotics were also able to inhibit the bacteria growth in disk diffusion assays as depicted in Figure 2 and Table 2.

**Figure 4:**
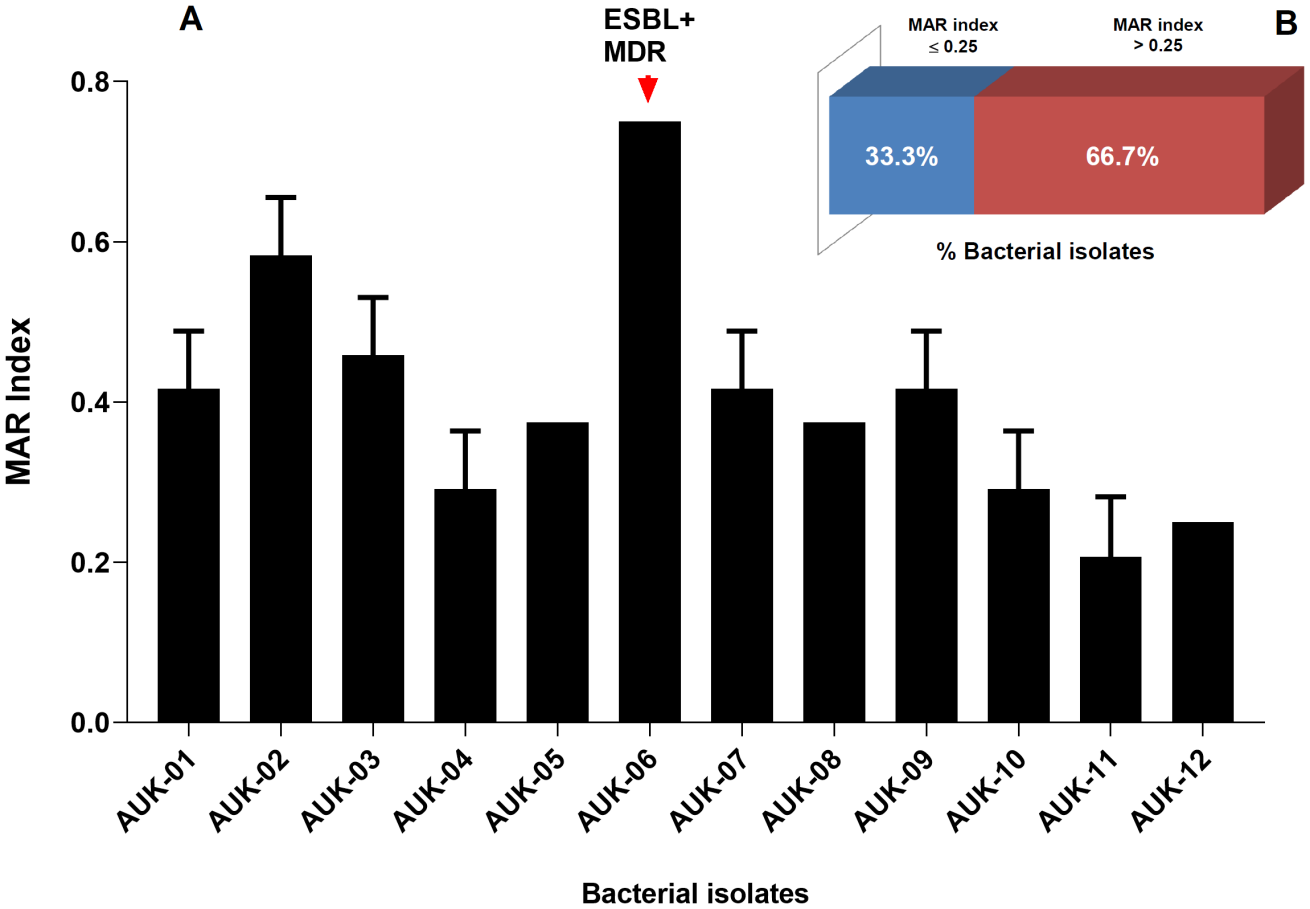
Multiple Antibiotic Resistance (MAR) index of bacteria isolated from irrigation-purpose wastewater samples from lower Himalayan agro-ecosystems located in Himachal Pradesh, India. MAR index was calculated according to the formula described by Krumperman (1983). Here, **(A)** represent the MAR indices of bacterial isolates and **(B)** represent the percentage of bacterial isolates having a MAR index ≤0.25 or > 0.25. Data represents mean ± SD of three independent experiments.

Phenotypic determination assay for ESBLs production revealed that six bacterial isolates namely AUK-01, AUK-02, AUK-04, AUK-06, AUK-08 and AUK-09 were ESBL-positive which represents half of the total isolates (Table 2). Among these, two isolates were *Enterobacter* spp., two were *E. coli* and one each was related to *Shigella* sp. and *Citrobacter* sp. Isolate AUK-06 (*Enterobacter* sp.) which was prevalent in wastewater sample of Solan city had exhibited both MDR and ESBL phenotypes. MAR index values for bacterial isolates ranged from 0.20 ± 0.07 to 0.75 (Figure 4A). The highest MAR index was exhibited by isolate AUK-06 (*Enterobacter* sp.) followed by isolate AUK-02 (0.58 ± 0.07) and AUK-03 (0.45 ± 0.07). The lowest MAR index was associated with isolates AUK-10, AUK-11 and AUK-12 which correlates with their low antibiotic resistance. In terms of percentage of bacterial isolates having a MAR index value of >0.25 was 66.7% whereas only 33.3% isolates had MAR index ≤0.25 (Figure 4B). These data indicated that the bacterial isolates originated from high antibiotic contamination aquatic environments.

**Figure 5:**
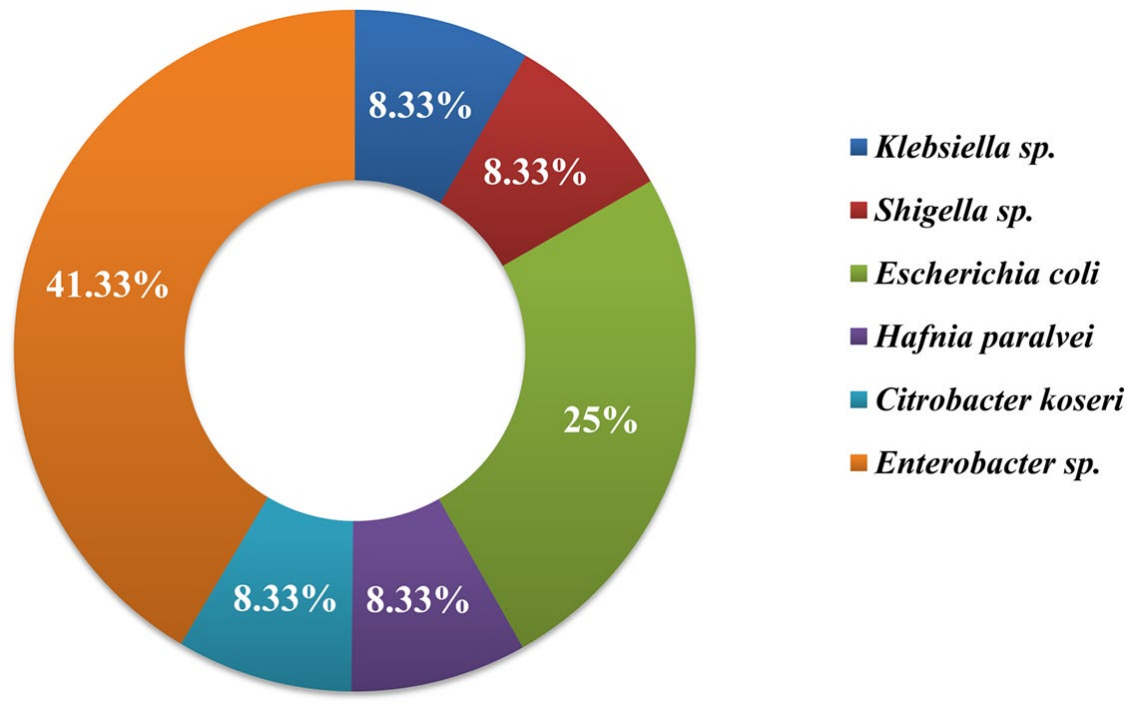
Genera-wise distribution (%) of bacteria (*n* = 12) isolated from irrigation-purpose wastewaters from lower Himalayan agro-ecosystems located in Himachal Pradesh, India.

**Figure 6:**
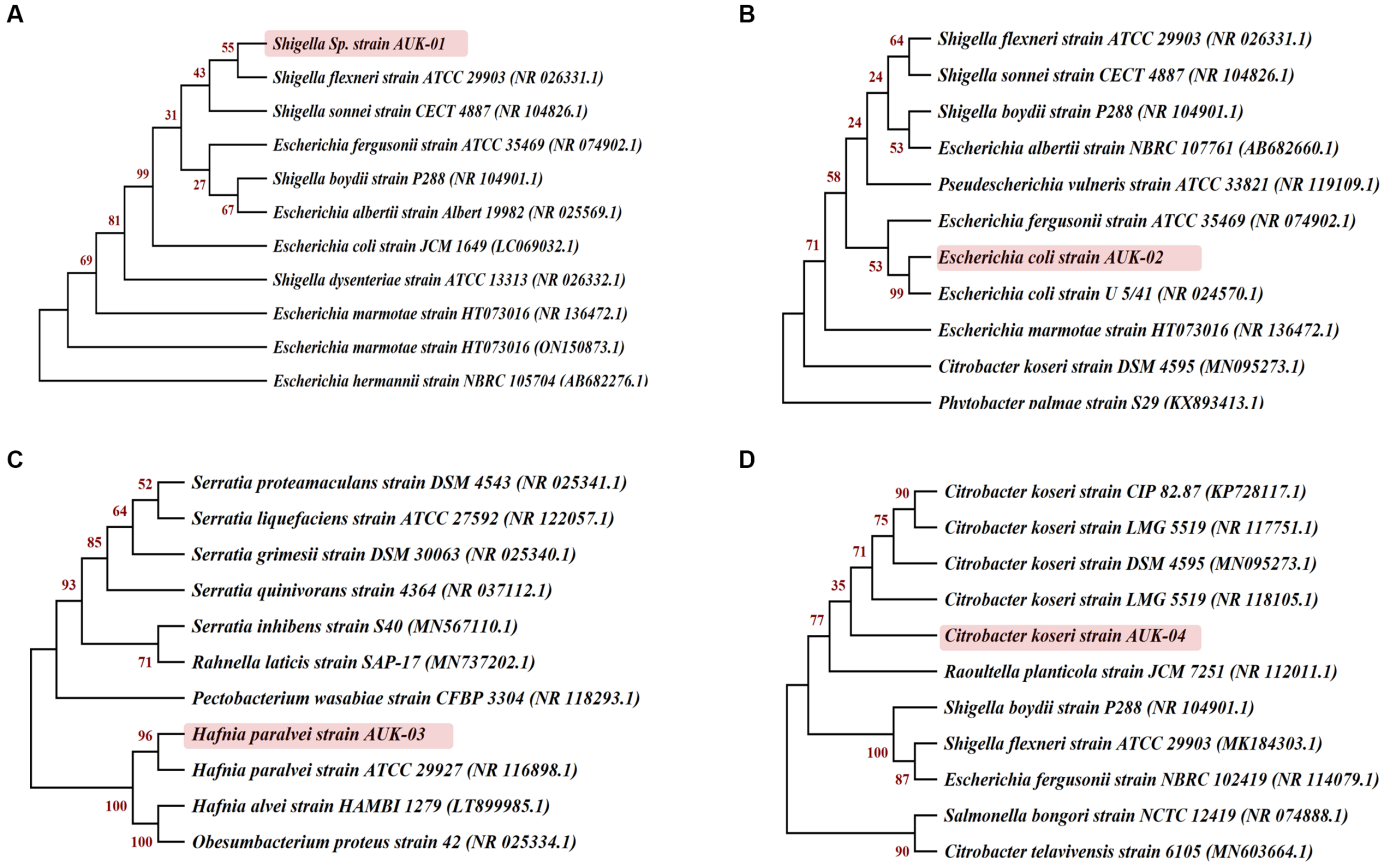
Phylogenetic relationships of selected bacterial isolates obtained from irrigation-purpose wastewater samples were inferred on the basis of 16S rRNA gene sequences by the neighbor-joining method using MEGA X software (version 10.2.5). Bootstrap values expressed as a percentage of 1,000 re-samplings are shown at the nodes. Here **(A)**, **(B)**, **(C)** and **(D)** represent the isolates AUK-01, AUK-02, AUK-03, and AUK-04, respectively.

Morphological, biochemical, and 16S rRNA gene sequence-based molecular characterization of Gram-negative bacterial isolates showed the dominant prevalence of *Enterobacter* sp. (41.3%) and *E. coli* (25%) as depicted in Figure 5. On the other hand, prevalence of *Klebsiella* sp., *Citrobacter koseri*, *Hafnia paralvei,* and *Shigella* sp. was low, i.e., 8.33% in wastewater samples. Four bacterial isolates, AUK-01, AUK-02, AUK-03, and AUK-04, were characterized by 16S rRNA gene sequencing and were phylogenetically related to *Shigella* sp., *Escherichia coli*, *Hafnia paralvei,* and *Citrobacter koseri*, respectively (Figure 6). The 16S rRNA gene sequences of these isolates were deposited in the NCBI GenBank database with the following accession numbers: ON968448, ON968449, ON968450, and ON968451. Further, the phylogenetic trees were constructed to assess the evolutionary relatedness among the bacterial isolates and their nearest neighbors available in the database (Figure 6).

## 4. Discussion

Wastewater contains high nutrient contents and hence found re-usability in agricultural irrigation systems since it provides organic carbon, nutrients (nitrogen, phosphorus, potassium), and inorganic micronutrients to the crop plants (Alcalde-Sanz and Gawlik, 2017; Minhas et al., 2022). The untreated wastewaters are frequently utilized for irrigation purposes in regions where rain shortfall are common or groundwater availability is limited which also include several states of India, including Himachal Pradesh. Their use in agricultural practices, however represent a serious health risk due to its inherent nature of carrying ARB and ARGs (Wang et al., 2020; Tucker et al., 2022). The antibiotic contents present in wastewaters facilitate mutation and genetic modifications in ARB thus making these multi-drug resistant and difficult to treat under clinical settings (Leiva et al., 2021; Uluseker et al., 2021; Mutuku et al., 2022). In the present study, 12 bacterial isolates belonging to six genera were identified in irrigation-purpose wastewater samples on the basis of morphological, biochemical, and molecular methods from 11 different geographical locations in Sirmaur and Solan districts of Himachal Pradesh. These bacteria belonged to *E. coli*, *Enterobacter* sp., *Hafnia* sp., *Shigella* sp., *Citrobacter* sp., and *Klebsiella* sp. *Enterobacter* spp. and *E. coli* were found to be most dominant among 12 bacterial isolates, respectively. The antibiograms of these isolates against penicillins (ampicillin and amoxyclav), aminoglycosides (gentamicin and tobramycin), sulfonamides (co-trimoxazole), and third-generation cephalosporins (cefotaxime, cefpodoxime, and ceftazidime) indicated resistance against all antibiotics except gentamicin. All isolates were resistant to ampicillin. In contrast, gentamicin exhibited antimicrobial action against all the isolates as all were susceptible to this antibiotic. Further, 11 isolates were resistant to amoxyclav whereas six of the isolates displayed resistance to cefotaxime. These findings are supported by previous research reports in which widespread resistance against similar antibiotic classes was reported in bacteria from *Enterobacteriaceae*. A high frequency of resistance to ciprofloxacin, tetracycline, cefoxitin, amoxicillin/clavulanic acid, cefotaxime, and aztreonam was reported from water samples of the Mondego River in Portugal (Amador et al., 2015). Schmiege et al. (2021); Zagui et al. (2020); Veloo et al. (2022) also observed MDR and ESBL-producing ARB in urban wastewaters. MAR index values for 12 isolates ranged between 0.21–0.75. Ali et al. (2021) found the MAR index value to range between 0.2 and 0.32 in wastewaters from Delhi-NCR region. The MAR index value >0.2 suggest high pollution load and antibiotic exposure in a specific sampling site (Krumperman, 1983; Ali et al., 2021).

Five isolates (AUK-05, AUK-06, AUK-07, AUK-08, and AUK-12) were identified to be *Enterobacter* spp. which showed their predominant prevalence in irrigation-purpose wastewater samples of the sampling sites. AUK-6 which was obtained from wastewater sample of Solan City was the sole MDR isolate of our study and it also showed ESBL phenotype. Similar to our findings, Ben Said et al. (2015) found the presence of the multidrug-resistant ESBL-producing *Enterobacter hormaechei* in irrigation water samples from Tunisia. In this study, 50% of bacterial isolates showed ESBL phenotypes, as confirmed by cefotaxime and cefotaxime/clavulanic acid combination-based qualitative methods. Among these isolates, AUK-01, AUK-02, AUK-04, AUK-06, AUK-08, and AUK-09 were ESBL producers. AUK-02 and AUK-09 were identified as *E. coli,* whereas AUK-06 and AUK-08 were related to *Enterobacter* spp. Further, *Shigella* sp. (AUK-01) and *Citrobacter* sp. (AUK-04) also displayed the ESBL phenotype. Ali et al. (2021) found 106 ESBL-positive isolates (24.3%) out of 436 bacterial isolates isolated from Hauz Khas lake, Ghazipur slaughterhouse, Jasola wastewater treatment plant, and Lodhi garden pond in the Delhi-NCR region. Among these isolates, 42–78% also exhibited MDR phenotypes, depending upon the location. Similarly, Singh et al. (2021) found ESBL-producing strains of *E. coli* (*n* = 34) and *K. pneumoniae* (*n* = 39) with a prevalence of *bla_CTX-M-1_, bla_CTX-M-2_,* and *bla_CTX-M-15_* genotypes from Manali, Kullu, and Baddi in Himachal Pradesh. In previous studies, antibiotic-resistant strains of *E. coli, Enterobacter, Klebsiella, Pseudomonas, Citrobacter, Hafnia, Acinetobacter, Shigella,* and *Aeromonas* have been reported from different regions of India (Lamba and Ahammad, 2017; Chaturvedi et al., 2020, 2021). The prevalence of the ESBL phenotype in 58% of *E. coli* isolates in irrigation water samples from several provinces of Ecuador was observed (Montero et al., 2021). Similarly, 33% Gram-negative bacterial isolates exhibited MDR phenotypes and ESBL genotypes (*bla*_KPC_, *bla*_TEM_, *bla*_SHV_ and *bla*_CTX-M_) from hospital sewage and urban wastewater in Brazil (Zagui et al., 2020). Igbinosa et al. (2023) reported that 85.9% of *E. coli* strains isolated from agricultural farms (irrigation water, soil, manure) were resistant to various antimicrobial classes and exhibited MDR phenotype as well ESBL genotypes which might be due to the use of untreated water for irrigation purposes. Tiwari et al. (2023) showed the presence of β-lactamase (*bla_GES_, bla_MOX_* and *bla_TEM_*) producing *Enterobacter* spp., *Aeromonas* spp., and *Klebsiella* sp. and *E. coli* in untreated municipal wastewater from Helsinki, Finland.

There are two ways that antibiotics can infiltrate agricultural ecosystems: firstly, by fertilizing with animal manures, biosolids, sewage sludge, and sediments that include antibiotics, and secondly, by irrigating with reclaimed water that has been contaminated with antibiotics from sewage treatment plants, wastewater, surface water, or groundwater since these sources are regularly contaminated with antibiotics (Du and Liu, 2012; Leiva et al., 2021; Minhas et al., 2022). In aquatic ecosystems, pathogenic, commensal, and environmental bacteria serve as reservoirs of antibiotic-resistance genes, mobile genetic elements, and bacteriophages, with consequent dissemination through conjugation, transformation, and transduction (von Wintersdorff et al., 2016). Utilization of wastewater for agricultural irrigation may assist in mobilizing clinically relevant antibiotic resistance genes (resistome) from non-pathogenic commensal bacteria to pathogenic strains, thus leading to the spread of resistance. The origin of *bla_CTX − M_* genes in *Enterobacteriaceae* can be traced to the environmental *Kluyvera* sp. via HGT (Canton and Coque, 2006). Therefore, efficient treatment technologies are essentially required for the removal of microbial pathogens, antibiotic residues and heavy metal hazards from wastewaters (Tucker et al., 2022). Regular monitoring and surveillance of antibiotic residues and bacteria resistant to antibiotics in wastewaters, rivers, ponds and wastewater treatment plants, and effluent treatment sites should be prioritized and included in regulatory action plans.

## 5. Conclusion

Wastewaters are being used in agricultural irrigation in several water-scarce countries due to their organic and inorganic nutrients, however this wastewater-based irrigation practices also present serious public health and environmental sustainability concerns. Our findings also confirmed the prevalence of antibiotic resistant Gram-negative bacteria resistant to multiple antibiotics and exhibiting the ESBL phenotype in irrigation-purpose wastewater samples from Himachal Pradesh. Therefore, there is an urgent need for an integrated approach focusing on environmental monitoring, identifying key hotspots of antibiotic resistance, early and rapid detection, continuous surveillance, stringent regulatory guidelines and improved agri-environmental risk assessment models before their use in the agro-ecosystems for ensuring public health and environmental safety.

## Data availability statement

The datasets presented in this study are deposited in the NCBI repository, accession numbers ON968448, ON968449, ON968450, ON968451: https://www.ncbi.nlm.nih.gov/nuccore/ON968448, https://www.ncbi.nlm.nih.gov/nuccore/ON968449, https://www.ncbi.nlm.nih.gov/nuccore/ON968450, and https://www.ncbi.nlm.nih.gov/nuccore/ON968451.

## Author contributions

AA, ShiS, and SK: conducted the experiments, and manuscript preparation. YA, SheS, DJ, PC, KP, MK, and NS: editing, review, and finalization of the manuscript. All authors contributed to the article and approved the submitted version.

## Conflict of interest

The authors declare that the research was conducted in the absence of any commercial or financial relationships that could be construed as a potential conflict of interest.

## Publisher’s note

All claims expressed in this article are solely those of the authors and do not necessarily represent those of their affiliated organizations, or those of the publisher, the editors and the reviewers. Any product that may be evaluated in this article, or claim that may be made by its manufacturer, is not guaranteed or endorsed by the publisher.

## Footnotes

^1^ http://blast.ncbi.nlm.nih.gov/Blast.cgi?PROGRAM=blastn
