# Supplementary material for: Prevalence of antibiotic-resistant Gram-negative bacteria having extended-spectrum β-lactamase phenotypes in polluted irrigation-purpose wastewaters from Indian agro-ecosystems
Source: Front Microbiol. 2023 Aug 7;14:1227132. doi: 10.3389/fmicb.2023.1227132 (PMC10440439; doi:10.3389/fmicb.2023.1227132)
Supplement: Supplementary file 1 [file Data_Sheet_1.PDF]

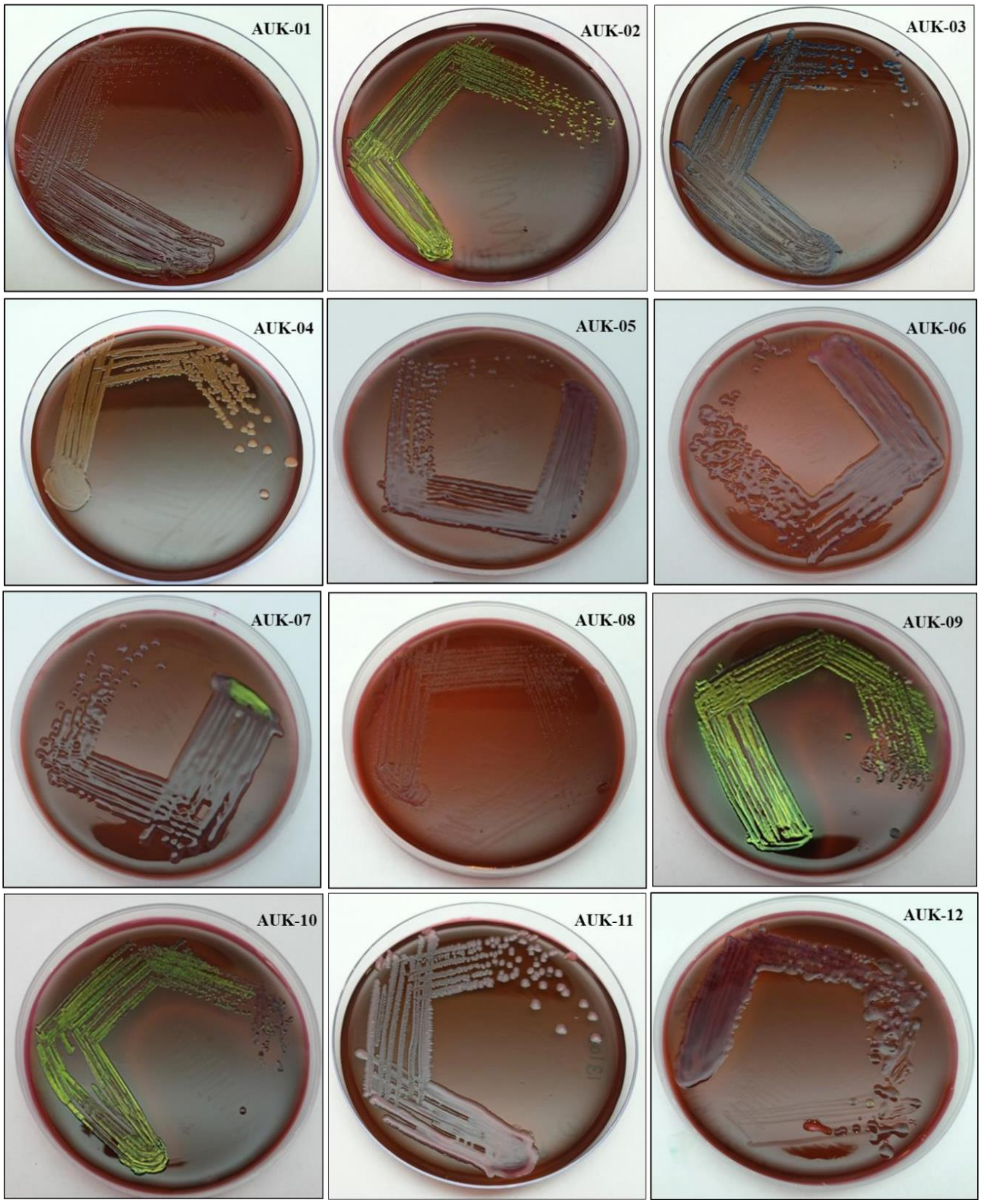

Fig. S1 The colony characteristics of pure cultures of bacterial isolates on EMB agar medium after 24 h incubation at 35-37°C under aerobic conditions.

Table S1 The colony characteristics, cell shape, arrangements and Gram staining profile of bacterial isolates obtained from wastewater samples.

S. No.	Isolate	Colony size	Colony shape	Colony Color/feature	Colony elevation	Colony texture	Gram's reaction	Cell shape	Cells arrangement
1.	**AUK-01**	Small	Circular	Pink	Convex	Smooth	Gram–ve	Bacilli (rod)	Single
2.	**AUK-02**	Small	Circular	Metallic green sheen	Convex	Smooth	Gram–ve	Bacilli (rod)	Single
3.	**AUK-03**	Medium	Circular	Blue	Convex	Smooth	Gram–ve	Bacilli (rod)	Diplobacilli
4.	**AUK-04**	Medium	Circular	Light brown	Convex	Smooth	Gram–ve	Bacilli (rod)	Single
5.	**AUK-05**	Medium	Irregular	Purple	Convex	Smooth	Gram–ve	Bacilli (rod)	Diplobacilli
6.	**AUK-06**	Medium	Irregular	Purple	Convex	Smooth	Gram–ve	Bacilli (rod)	Single
7.	**AUK-07**	Medium	Irregular	Purple	Convex	Smooth	Gram–ve	Bacilli (rod)	Single
8.	**AUK-08**	Small	Circular	Pink	Convex	Smooth	Gram–ve	Bacilli (rod)	Single
9.	**AUK-09**	Small	Circular	Metallic green sheen	Convex	Smooth	Gram–ve	Bacilli (rod)	Single
10.	**AUK-10**	Small	Circular	Metallic green sheen	Convex	Smooth	Gram–ve	Bacilli (rod)	Single
11.	**AUK-11**	Medium	Circular	Pink	Convex	Smooth	Gram–ve	Bacilli (rod)	Single
12.	**AUK-12**	Medium	Irregular	Purple	Convex, raised	Smooth	Gram–ve	Bacilli (rod)	Diplobacilli

Table S2 Biochemical characterization of bacterial isolates obtained from wastewater samples.

Isolate	Indole test	Methyl red	VP test	Citrate utilization	Glucose	Adonitol	Arabinose	Lactose	Sorbitol	Mannitol	Rhamnose	Sucrose	Oxidase	Catalase
**AUK-01**	+	–	–	–	+	–	–	+	–	–	+	–	–	+
**AUK-02**	+	+	–	–	+	+	+	+	–	+	–	+	–	+
**AUK-03**	–	+	+	+	+	–	+	–	–	+	+	–	–	+
**AUK-04**	–	+	–	+	+	–	–	+	–	–	–	–	+	+
**AUK-05**	+	–	+	+	+	–	+	+	–	+	–	+	–	+
**AUK-06**	+	–	+	+	+	–	+	–	+	+	–	+	+	+
**AUK-07**	+	–	+	+	+	–	+	+	–	+	+	+	–	+
**AUK-08**	+	–	+	+	+	–	+	+	–	–	–	+	+	**-**
**AUK-09**	+	+	–	–	+	–	+	+	+	+	+	–	–	+
**AUK-10**	+	–	–	+	+	+	+	+	+	+	+	+	–	+
**AUK-11**	–	+	+	+	+	+	+	+	+	+	+	+	–	+
**AUK-12**	+	–	+	+	+	–	+	–	+	+	–	+	+	+

VP = Voges-Proskauer‘s test
(–) negative result; (+) positive result
